# Extraction of Nanocellulose for Eco-Friendly Biocomposite Adsorbent for Wastewater Treatment

**DOI:** 10.3390/polym14091852

**Published:** 2022-04-30

**Authors:** Mohamed Bassyouni, Mohamed Sh. Zoromba, Mohamed H. Abdel-Aziz, Ibrahim Mosly

**Affiliations:** 1 Department of Chemical and Materials Engineering, King Abdulaziz University, Rabigh 21911, Saudi Arabia; mzoromba@kau.edu.sa (M.S.Z.); mhmossa@kau.edu.sa (M.H.A.-A.); 2 Department of Chemical Engineering, Faculty of Engineering, Port Said University, Port Said 42526, Egypt; 3 Chemistry Department, Faculty of Science, Port Said University, Port Said 42521, Egypt; 4 Chemical Engineering Department, Faculty of Engineering, Alexandria University, Alexandria 21544, Egypt; 5 Department of Civil Engineering, King Abdulaziz University, Rabigh 21911, Saudi Arabia; ikmosly@kau.edu.sa

**Keywords:** nanocellulose, chitosan, microbeads, adsorption, isothermal models, direct dye removal

## Abstract

In the present study, nanocellulose was extracted from palm leaves to synthesize nanocellulose/chitosan nanocomposites for the removal of dyes from textile industrial wastewater. Nanocellulose is of interest in water purification technologies because of its high surface area and versatile surface chemistry. Following bleach, alkali, and acid treatments on palm leaves, nanocellulose is obtained as a white powder. The produced nanocellulose was investigated. The adsorption capacity of chitosan, nanocellulose, and novel synthetic nanocellulose/chitosan microbeads (CCMB) for direct blue 78 dye (DB78) removal was studied. A series of batch experiments were conducted in terms of adsorbent concentration, mixing time, pH, dye initial concentration, and nanocellulose concentration in synthetic microbeads. The CCMB was characterized by using physicochemical analysis, namely Brunauer–Emmett–Teller (BET), scanning electron microscope (SEM), zeta potential analysis, and Fourier-transform infrared spectroscopy (FTIR). It was found that the surface area of synthetic CCMB is 10.4 m^2^/g, with a positive net surface charge. The adsorption tests showed that the dye removal efficiency increases with an increasing adsorbent concentration. The maximum removal efficiencies were 91.5% and 88.4%, using 14 and 9 g/L of CCMB-0.25:1. The initial dye concentrations were 50 and 100 mg/L under acidic conditions (pH = 3.5) and an optimal mixing time of 120 min. The equilibrium studies for CCMB-0.25:1 showed that the equilibrium data were best fitted to Langmuir isothermal model with R^2^ = 0.99. These results revealed that nanocellulose/chitosan microbeads are an effective eco-adsorbent for the removal of direct blue 78 dye and provide a new platform for dye removal.

## 1. Introduction

Dye contaminants in aquatic resources have become a significant problem as a result of the recent industrialization, urbanization, and growth of dyes-based operations such as the textile industry. Textile industries are also one of the world’s fastest-growing industrial sectors presently. They use a lot of water and produce a large amount of wastewater, which mostly consists of colors used in the dyeing process. The yearly water consumption by textile industries in the world is 40 × 10^9^ m^3^ [1]. Approximately 10–15% of the applied dyes amount is gushed out as effluent. Such wastewaters severely affect land and water, resulting in pollution of the ecosystem [2,3].

The organic effluent disrupts the aquatic biosphere by obstructing light penetration, as well as posing major health risks to humans [4,5,6,7]. These dangers serve as ongoing reminders of the importance of finding effective color-removal technologies for wastewater. Many treatment technologies specialized in dye removal have been investigated, with several degree of success (i.e., chemical coagulation, membrane separation, catalytic, and chemical/physical adsorption) [8,9,10,11]. Among these processes, adsorption is considered one of the most commonly utilized and adaptable, allowing for the efficient and cost-effective removal of pollutants [12,13,14,15,16]. It was reported that biosorption process is one of the most promising technologies for wastewater treatment. Microorganisms such as bacteria, fungi, and algae have been investigated and used efficiently as bio-based sorbents for the removal of many contaminates from wastewater [17,18,19].

Various dyes adsorbents have been designed and examined, such as graphene-based composite, activated carbons, inorganic nanomaterials, microorganisms, and metal–organic frameworks.

Because of their natural abundance, low cost, biocompatibility, and low environmental impact, biomass materials such as cellulose, chitin, chitosan, and lignin have been recognized as one of the most promising candidates in the past few years [20,21].

Cellulose is the most important plant component to substitute synthetic polymers, due to its being an inexpensive, nontoxic, and biodegradable polymer [22]. Cellulose is the main component of the plant cell wall [23,24]. Cellulose can be extracted from various sources, including wood, grasses, seed fibers, date palm seeds, algae, sisal fibers, fungi, argo-industrial waste, and bacteria [25,26,27]. On the other hand, cellulose is the most plentiful and renewable naturally occurring polymer in the world. It has been used in the manufacturing of energetic material for numerous supplies in the food and pharmaceutical industries, in paint, and in textiles [25]. In recent years, a wider application of cellulose has been suggested at the nanoscale level for developing various biocompatible products and a variety of cellulose derivatives [28,29,30]. A new area of nanocellulose applications is still under examination in some fields, including photonics, foams, surface modifications, nanocomposites, flexible, pharmaceutical industries, and optoelectronics. The most useful property of nanocellulose exploration is the green nature of the particles, as well as amazing chemical and physical properties. In addition, there are a variety of applications that can be taken from this vital material [31]. Cellulose is a carbohydrate polymer that has many monosaccharide units that are connected to each other through covalent bonds. Cellulose is considered to be a polymer with a linear backbone of anhydro-glucose monomer units connected through 1,4 β-linkages. Moreover, cellulose’s compact structure, mainly contributed to by the linkage of intra- and inter-hydrogen bonds as strong physical bonds, has a remarkable mechanical strength that protects the plant’s biological structure [32]. Date palm fiber biomass is a potential renewable resource which can contribute to energy sustainability. This may diminish the negative impacts of petroleum combustion in the environment. In the production of nanocellulose material from lignocellulosic biomass, various methods have been used, including acidic, basic, and ionic liquid treatment [33,34,35]. During the biomass treatment process, potassium hydroxide solution makes polymers (hemicellulose, lignin, and cellulose) swell, partially breaking the intra-hydrogen bonding in the biomass structure. The less ordered biomass structure leads to an increase in the number of existing hydroxyl groups and the availability of solvents for further hydrolysis reaction. Usually, sulfuric acid or hydrochloric acid as a strong acid were used for de-polymerization processing; acid makes hydrolytic cleave to the glycosidic linkages between the two adjacent anhydroglucose nits, dissolving the amorphous region of the cellulose by increasing the crystallinty of cellulose [36]. Because contamination of water by toxic dyes can affect human health and the ecology, it is necessary to remove dye wastewater. Natural adsorbents have attracted the attentions of many researchers throughout the world, due to their availability, ease of modification, and excellent adsorption capability. Heavy metals and dyes are removed from wastewater by using cellulose nanofibers (CNFs) [37]. This crystalline portion may be isolated from cellulose fibrils, which are typically 50–150 nm in length [38]. Nanocellulose (NC) may be versatilely modified with other materials to improve its adsorption capacity in the presence of hydroxymethyl functional groups. Some studies have found that using nanocrystalline cellulose (NC) to remove anionic and cationic dyes has satisfactory results [39]. More than 300 million tons of paper is produced yearly, and the demand is expected to increase by 2030 [40]. Paper uses 42 percent of the world’s wood, generating environmental issues.

It was reported that a tris-azo dye (Direct Blue 71) was removed from aqueous solutions by using chitosan-based adsorbent gels. The chemical modification of chitosan was carried out by using crosslinking agents: sodium tripolyphosphate (TPP) and glutaraldehyde. The linear and nonlinear approaches for the Langmuir and Freundlich isotherms were compared. The adsorption studies revealed that the Langmuir model best described the experimental data from this study, with the maximal dye adsorption capacity of the adsorbent being 88.49 mg/g (linear form) and 92.22 mg/g (square form) (nonlinear form) [41,42].

**Figure 1:**
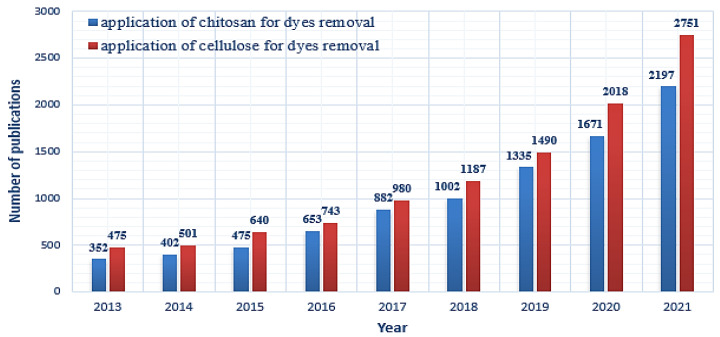
Published papers related to utilization of chitosan and cellulose for dye removal. Obtained from ScienceDirect. Search words, respectively, were chitosan adsorption for dye removal and cellulose adsorption for dye removal.

Chitosan hydrogel beads were made by lowering the degree of crystallinity by generating a gel with the purpose of raising chitosan’s adsorption capability. Several strategies, including chemical crosslinking with crosslinking agents on their surface, have been developed to improve the commercial applicability of chitosan beads [43,44,45,46,47,48]. In recent years, the utilization of chitosan and cellulose for dye removal has attracted substantial interest as a potential eco-friendly adsorbent. Figure 1 shows the number of yearly published articles from 2013 to 2021. It was noted that the number of scientific publications on chitosan and cellulose in dye removal have significantly increased over the last 9 years.

This study aimed to extract nanocellulose from palm leaves and develop a novel biocomposite adsorbent from the two largest natural resources (nanocellulose and chitosan), with an ionic liquid as the medium. The adsorption efficiency for direct blue 78 dye (anionic) removal using chitosan, nanocellulose, and nanocellulose/chitosan biocomposites was investigated.

In this research, the nanocellulose particles were successfully extracted from palm leaves. A novel nanocellulose-based adsorbent was used for direct blue 78 dye removal with high efficiency (88–91%), optimal dose = (9–14) g/L, and high sedimentation rate, with an initial dye concentration of 50 and 100 mg/L respectively. Isothermal studies were also conducted for nanocellulose-based adsorbents, and the findings showed that the Langmuir isotherm best fit the adsorption results (R^2^ = 0.98).

## 2. Materials and Methods

### 2.1. Extraction of Nanocellulose from Palm Fiber

**Figure 2:**
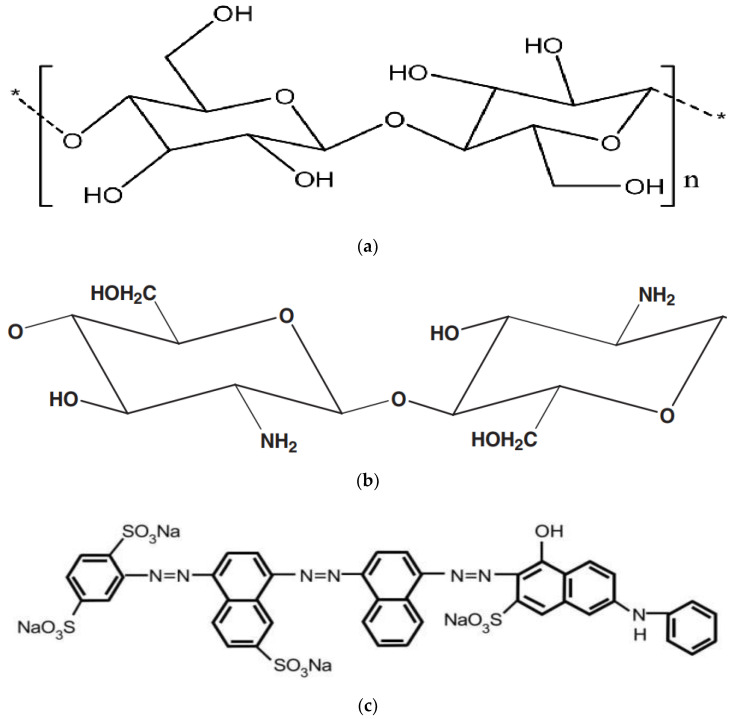
(**a**) Chemical structure of nanocellulose. (**b**) Chemical structure of chitosan. (**c**) Chemical structure of direct blue 78 dye.

Palm leaves were cleaned, shredded, and ground. Acetic acid, sodium hydroxide and sodium chlorite were purchased from Sigma-Aldrich (St. Louis, MI, USA). A solution of acetic acid, sodium chlorite, and distilled water was used for de-lignification. The suspension solution was refluxed at 80 °C. The solution was stirred for 24 h at 65 °C before rinsing with deionized water. The separation process was carried out by using centrifugation at 8000 r.p.m. The holocellulose was obtained at 70 °C. The remaining materials (hemicellulose and lignin) were removed by using diluted sodium hydroxide, using magnetic stirring for 7 h. The resultant suspended solution was dried for 24 h at 65 °C. Nanocellulose was extracted by using sulfuric solution (9 M) after stirring for 7 h at ambient temperature. The solution was rinsed with distilled water to avoid cellulose hydrolysis. Nanocellulose was obtained after centrifugation and freezing. Cellulose is a linear homopolymer made up of repeating units (called cellobiose) that are formed by connecting two anhydro-glucose rings via a -1,4 glycosidic linkage. It has s abundance of hydroxyl groups on the surface, according to its structure, as shown in Figure 2a.

### 2.2. Chitosan and Dyes

Figure 2b shows chitosan, an amino-based polymer, synthesized in vast amounts by N-deacetylation of chitin. High-molecular-weight chitosan was used. The supplier reported that it is a white powder with a molecular-weight range from 140 to 220 kDa, degree of deacetylation (DAC) of 81.2%, viscosity of 36,000 cps, and density of 0.15 g/mL.

The direct blue 78 (DB78) dye (Port Said, Egypt)was received by color print (Port Said, Egypt). Its relative molecular mass was 1059.95, max wavelength (λ_max_) was 604 nm, and solubility was up to 10 g/L at 25 °C. The direct blue 78 was selected for adsorption tests, as it is widely used in the textile industry. The chemical structure of direct blue 78 is shown in Figure 2c. Two synthetic dye solutions with different dye concentration (50 and 100 mg/L) were prepared for adsorption study.

### 2.3. Preparation of Nanocellulose/Chitosan Microbeads (CCMB)

**Figure 3:**
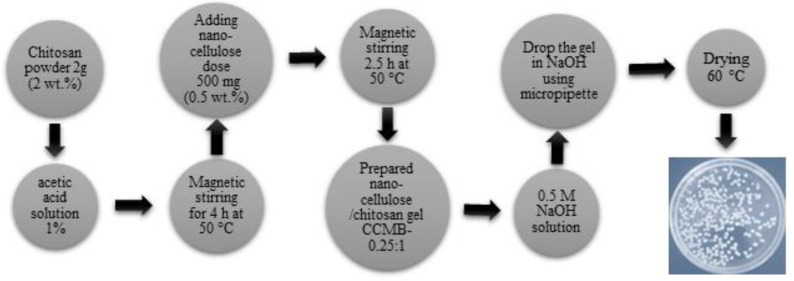
The preparation process for nanocellulose/chitosan beads (CCMB-0.25:1).

Chitosan was purchased from Sigma-Aldrich (St. Louis, MO, USA). Nanocellulose/chitosan microbeads (CCMBs) with different ratios of nanocellulose to chitosan were synthesized: CCMB_z_, where z refers to the ratio of nanocellulose to chitosan. The adsorption studies were conducted by using polymer biocomposite materials with different loading ratios: CCMB-0.1:1, CCMB-0.25:1, CCMB-0.5:1, and CCMB-1:1. In CCMB-1:1, the chitosan solution was prepared under magnetic stirring for 4 h by dissolving 2 g (2 wt.%) of chitosan powder into diluted acetic acid to form a 100 mL chitosan gel sample. A total of 500 mg (0.5 wt.%) of nanocellulose was added to the formed gel (50 mg of nanocellulose was added for every 10 mL of chitosan gel, under magnetic stirring, for 2.5 h, at a temperature of 50 °C). The final prepared gel was dropped into a 0.5 M NaOH solution (contact time 6 h), using a micropipette to form the beads. The formed beads were then washed with distilled water. Finally, the beads were oven-dried at 60 °C. The preparation process is illustrated in Figure 3.

### 2.4. Adsorption Studies

This study was conducted by using a discontinuous batch adsorption system (lab scale) on a single-component synthetic wastewater. Nanocellulose samples used in this study were 0.1–2 g in 1000 mL of synthetic wastewater, with differing initial concentrations (50 and 100 mg/L), with mixing at 150 r.p.m. and contact times of 0–60 min, at a room temperature (22 ± 2 °C). Chitosan samples used in this study were 1–6 g in 1000 mL of synthetic wastewater, with differing initial concentrations (50 and 100 mg/L), with mixing at 150 r.p.m. for contact times of 0–60 min, at a room temperature (22 ± 2 °C). Nanocellulose/chitosan microbead samples used in this study were 1–15 g in 1000 mL of synthetic wastewater with differing initial concentrations (50 and 100 mg/L), with mixing at 150 r.p.m. for different contact times (0:150 min), at a room temperature (22 ± 2 °C). The spectrophotometrically analysis was applied to determine the removal efficiency by measuring dyes’ concentrations before and after the adsorption process at λ_max_ = 600 nm for DB78.

#### Nanocellulose and Chitosan Nanocomposites

The nanocellulose suspension was diluted in water and ultrasonicated for 30 min in an ultrasonic bath, at 4% (*w*/*v*). Model USC-1400 is one-of-a-kind (40 kHz of ultrasound frequency). The Malvern 3000 Zetasizer NanoZS was used to make the measurements (Malvern Instruments, Malvern, WR14 1XZ. UK). This apparatus measures the diffusion of particles moving under Brownian motion and translates the data to size and size distribution, using dynamic light scattering. It also employs laser doppler micro-electrophoresis to provide an electric field to a dispersion of particles, which then move at a rate proportional to their zeta potential. The Smoluchowski algorithm was used to determine the particle size.

The surface area of the CCMB-0.25:1 sample was measured in the presence of N_2_ adsorption at −195.65 °C, using surface area analyzers (Autosorb-l-C-8, Quantachrome, Boynton Beach, FL, USA). Prior to adsorption studies, the samples were degasified at 200 °C for 4 h. By applying the BET (Brunauer–Emmett–Teller) equation to the adsorption data, the BET surface area for the sample was determined.

The colorimetric analysis was performed in this study, using a spectrophotometer (LAMOTTE smart spectrophotometer v3 2000-01-MN, Washington Ave. Chestertown, MD, USA).

The pH values of nanocellulose, chitosan, and CCMB-0.25:1 solutions were determined by mixing 0.1 g from each sample with 100 mL of distilled water, at a mixing speed of 100 r.p.m., for a period of 1 h and temperature of 25 °C, using a digital pH meter (Omega CDS107, Taiwan).

The surface morphology and porous microstructure of the CCMB-0.25:1 samples were investigated by SEM analysis, using a Quanta 250 FEG scanning electron microscope (Field Electron and Ion company, Hillsboro, OR, USA.).

FTIR studies for the nanocellulose, chitosan, and CCMB-0.25:1 samples were observed by using a VERTEX 80v vacuum FTIR Spectrometer (Bruker corporation, Oberkochen, Germany).

## 3. Results and Discussion

### 3.1. Characterization of Nanocellulose

The extracted nanocellulose was characterized by using zeta sizer to measure the particles size. Chemical structure was determined by using FTIR analysis.

#### 3.1.1. Size of Nanocellulose

**Figure 4:**
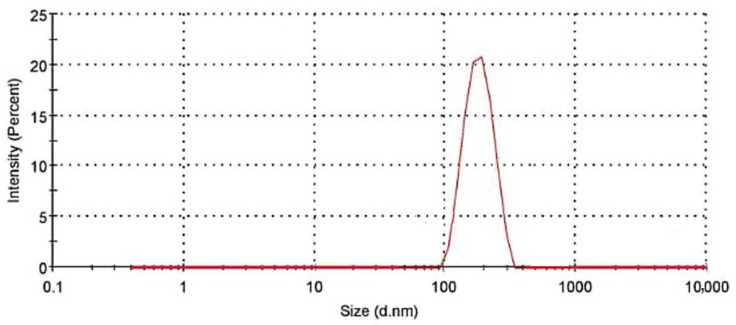
Particle size distribution of nanocellulose.

To generate hydrodynamic diameter dimensions, light-scattering data were automatically evaluated and computed by using the built-in Zetasizer program. Figure 4 illustrates the particle size distribution acquired from DLS; it demonstrates that 95.5 percent of particles fall between nano-dimensions (up to 300 nm).

#### 3.1.2. X-Ray Diffraction (XRD) Analysis

**Figure 5:**
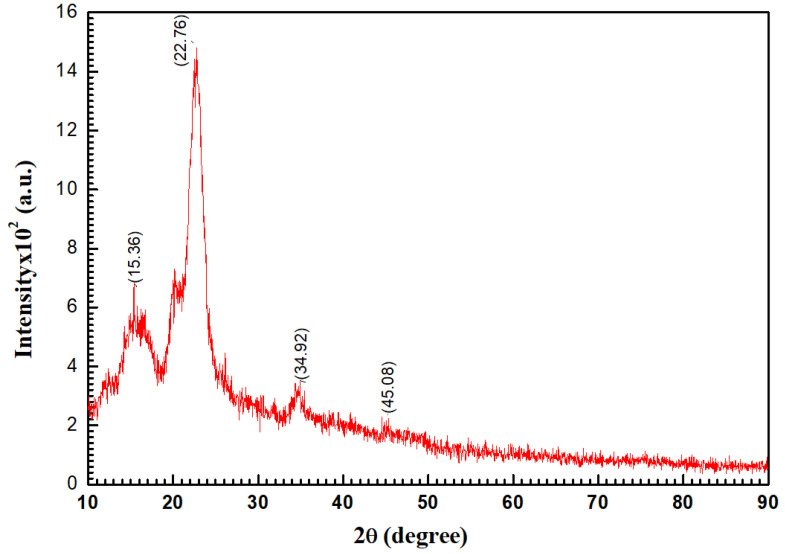
XRD pattern of cellulose sample.

Figure 5 shows the XRD pattern of the cellulose powder; the broad peaks indicate the amorphous nature of the cellulose powder. The XRD data (angle of diffracted beams, Miller indices (hkl), interplanar spacing, full width at half-maximum, and crystalline size (D) of cellulose powder) are listed in Table 1. Based on the diffraction peaks, a monoclinic 2 structure of cellulose was recorded with the following lattice parameters: *a* = 15.9634 Å, *b* = 7.85020 Å, *c* = 10.8664 Å, *α* = *γ* = 90, and *β* = 97.931°. The lattice parameters were calculated from the peak position, as given by the following relation [49]:

(1) $$\frac{1}{d^{2}}=\frac{1}{sin^{2}\theta }\left(\frac{h^{2}}{a^{2}}+\frac{k^{2}sin^{2}\beta }{b^{2}}+\frac{l^{2}}{c^{2}}-\frac{2hkcos\beta }{ac}\right)+\frac{l^{2}}{c^{2}}\;$$

As shown in Table 1, the estimated crystallite size (D) and miller index (hkl) are dependent on the absolute values of full width at half maximum (FWHM). The data in database code_amcsd 0,017,094 agree well with the interplanar distances’ d-spacing [50], according to the American Mineralogist Crystal Structure Database. The Debye–Scherrer method was applied to assessed XRD for cellulose powder, the range of 10 ≤ 2θ ≤ 90 with $1/dhkl=0.0566{Å}^{-1}-0.7446{Å}^{-1}$, λ=1.540562 Å, $I_{2}/I_{1}=0.5$, polarization = 0.5, and function Pseudo-Voigt. From Scherer’s formula, we obtain the following:

(2) $$D=\frac{0.9\lambda }{FWHM.cos\theta }\;$$

where *λ* is the X-ray wavelength (1.541838 Å). As presented in Table 1, for cellulose powder, the XRD data from the XRD pattern were used to examine factors and features such as FWHM, the crystallite size (D), hkl indices, d-spacing (d), and peak intensity. The crystalline size $D_{av}=164.71\;\mathrm{nm}$ was within the range of 83.04–183.56 nm for cellulose powder. While, for both the experimental and PXRD models, the intensity and location of specific peaks vary only slightly, the emphasis here is mostly on their overall resemblance. Only the important comparison characteristics between the measured and the experimental data should therefore be evaluated. It is also known that instrumentation and data-collection processes are only two of the many variables that can affect the experimental PXRD pattern. Employing X-ray powder diffraction to distinguish patterns of cellulose Iα and cellulose Iβ is exceedingly difficult, due to their overlap [51].

#### 3.1.3. Thermal Analysis of Nanocellulose

**Figure 6:**
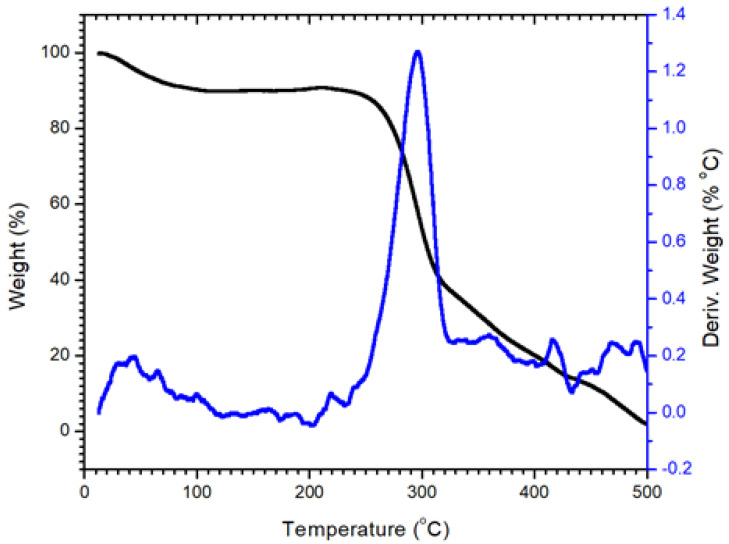
Thermal analysis TG and DrTGA for cellulose at 20–500 °C.

**Figure 7:**
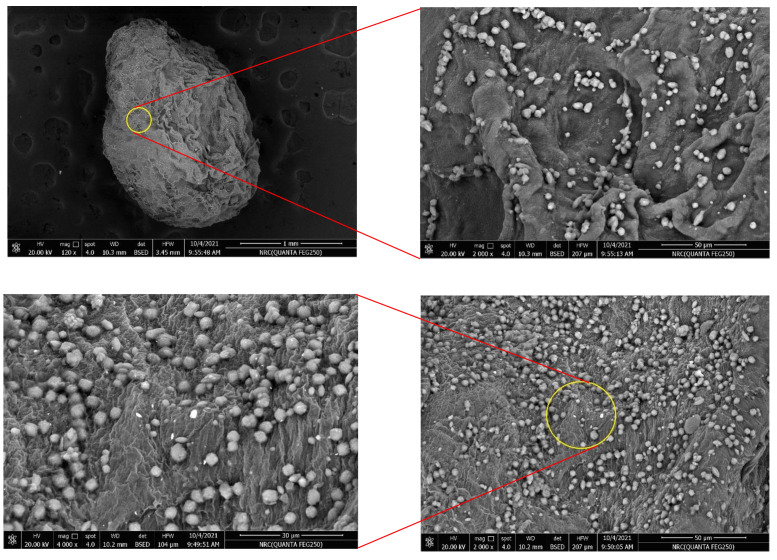
SEM image with different magnification for nanocellulose/chitosan microbead (CCMB-0.25:1).

The stability properties of cellulose are shown in Figure 6. At temperatures below 100 °C, moisture evaporation was observed in the cellulose samples. There was low weight loss within this stage because the amount of absorbed water or moisture in cellulose is low. Around 7% weight loss was recoded up to 100 °C. This process mostly relates to water moisture evaporation below 100 °C, as validated by the DrTGA investigation shown in Figure 7. It was found that cellulose breakdown began at 307 °C and lasted until 340 °C. These results are in a good agreement with the reported data [52]. The thermal stability of cellulose chains is enhanced by their highly ordered packing into systems (crystals) and by strong hydrogen bonding. The crystalline structure of cellulose is essential for its heat stability [53]. The observed narrow curve at DrTGA 307 °C might potentially be due to more surface area exposed to the heat and partial disruption when the temperature increased from 270 °C to 340 °C. Finally, the decomposition of cellulose was found from 330 to 500 °C. This stage can be attributed to cellulose oxidation. One medium peak can be found in the DrTGA curve at a temperature of 420 °C and refers to the decomposition of polymer chains of cellulose. A similar result was reported for nanocellulose decomposition using TGA at around 420 °C [54,55].

#### 3.1.4. FTIR Analysis

**Figure 8:**
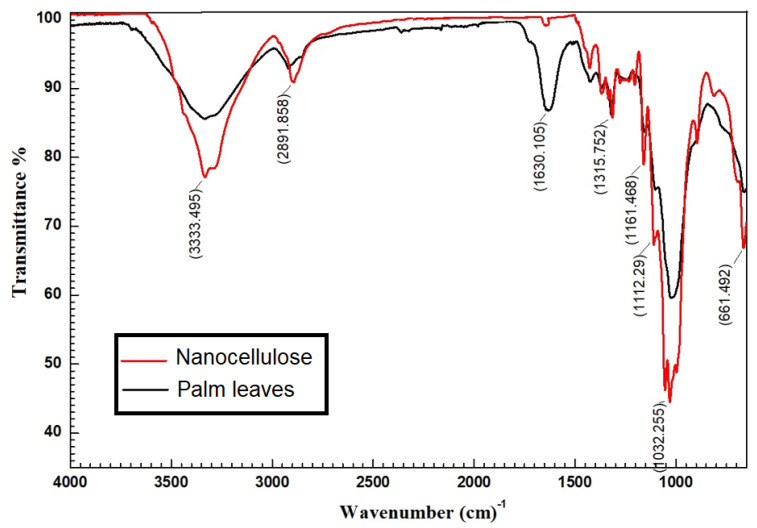
FTIR analysis of cellulose and palm leaves.

The FTIR results of cellulose and palm fibers are shown in Figure 8. The results show that the cellulose has a broadband at the 3432 cm^−1^ region that can be attributed to O-H groups’ stretching vibration. As shown in the spectrum, the bands appearing in the regions (1322–1429), (2997–3766), (1561–1806), and (626–843) cm^−1^ can be attributed to the hydroxide group of water molecule and may be bending, stretching, rocking, and wagging vibrations. This indicates the presence of water molecules in the studied copolymer [56]. There were several absorption bands that were associated with the cellulose, and we it was also observed that 1160 and 1062 cm^−1^ were attributable to C-O bond stretching [57]. The cellulose with abundant surface hydroxyl groups was investigated by using FTIR, as shown in Figure 8. The appearance of a new peak at 1161cm^−1^ is associated with C=C stretching [58]. The absorption peak noticed at 1630 cm^−1^ refers to the O-H bond of absorbed water.

The appearance of a new peak at 2354 cm^−1^ that was associated with ester groups was intensively observed on cellulose. Among the three kinds of OH groups, the OH group of the sixth position acts as a primary alcohol, where most of the modification predominantly occurs [59].

### 3.2. Characterization of Nanocomposites

#### Surface Morphology of Nanocomposites

Because of the importance of surface morphology and its great influence on the adsorption process, SEM analysis was investigated for CCMB-0.25:1 in order to identify its surface morphology and nanocellulose particles’ distribution on the beads’ surface. Figure 7 shows the SEM image for the nanocellulose/chitosan microbead (CCMB) surface. The SEM images showed that all nanocellulose particles were incorporated effectively into chitosan network, and there is no agglomeration of large numbers of nanoparticles the on small surface area. This efficient distribution of nanocellulose particles on the chitosan microbeads’ outer surface resulted in the creation of a large number of adsorption active sites. Moreover, it can be noticed from the SEM analysis that CCMB has an average particle size of 2 µm, with a large number of micropores.

The efficiency of the adsorption process is affected by the distribution of cellulose particles on the bead surface; with a uniform distribution of nanocellulose particles on beads’ surface, the adsorption behavior would be improved. The BET surface area for CCMB-0.25:1 was determined (SBET = 10.4 m^2^/g).

**Figure 9:**
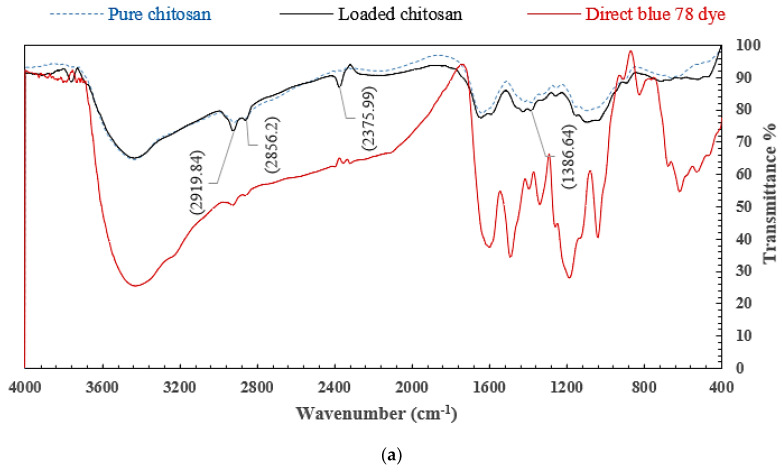

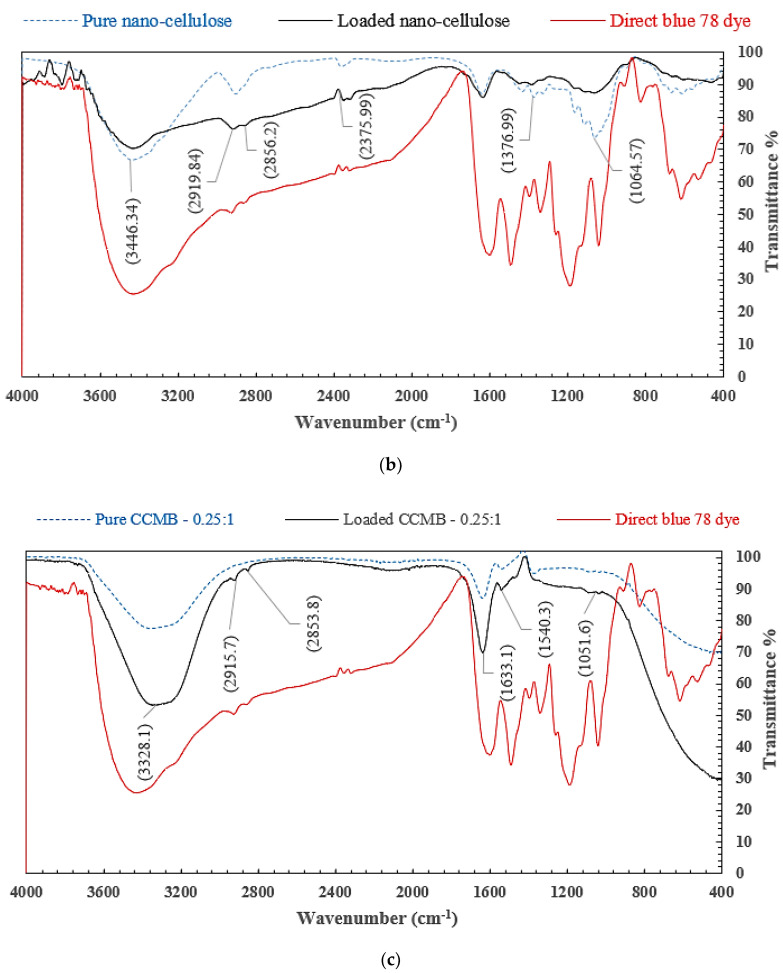
FTIR spectra for (**a**) pure and loaded chitosan, (**b**) pure and loaded nanocellulose, and (**c**) BD78 dye and CCMB-0.25:1 before and after adsorption process.

In order to classify the main infrared (IR) bands of organics and determinate the adsorption mechanism (physisorption or chemosorption), pure and loaded samples of chitosan, nanocellulose, and CCMB-0.25:1 were investigated by FTIR analysis. As shown in Figure 9a, the peaks at 2919 and 2856 cm^−1^ can be attributed to C-H symmetric and asymmetric stretching, respectively. The cH_2_ bending and cH_3_ symmetrical deformations were confirmed by the presence of a peak at 1386 cm^−1^. The new peaks that appeared in the FTIR spectra for loaded chitosan when compared with the pure chitosan are attributed to the chemical bond formed between dyes molecules and -NH_2_ groups on the surface of chitosan particles after the adsorption process. These results were also reported by Reference [60].

Figure 9b shows the FTIR analysis of nanocellulose before and after dye adsorption. The peaks at 3446 and 2919 cm^−1^ can be attributed to an O-H stretching band caused by the hydrogen-bonded hydroxyl group variations of cellulose and aliphatic saturated symmetric C-H stretching variations in cellulose, respectively. The presence of bands at 1660 cm^−1^ can also be caused by O-H bending modes of adsorbed water. The C-O-C band was also confirmed by the pretense of a peak at 1064 cm^−1^. When comparing the two spectra, we noticed that there is no difference. New peaks have not yet appeared, but the peak wave number shifted to higher values. That means that chemical bonds were not formed, and the adsorption process was conducted due to the electrostatic interaction between anionic dyes molecules and H^+^ ions accumulated on the nanocellulose surface in acidic conditions [61].

Figure 9c shows the FTIR spectra for pure and loaded CCMB-0.25:1. It was observed that there is a significant difference. This difference is attributed to the chemical bonds formed between -SO_3_ groups on dye molecules and -NH_3_^+^ groups on the beads’ surface.

The most relevant difference is the appearance of bands at 2915 and 2853 cm^−1^. These bands originated from dye molecules attached to the beads’ surface after adsorption process. Figure 9c also shows the -OH stretching vibration, which can be represented by the peak at 3328 cm^−1^. The stretching frequency of the –NH_2_ groups can be seen from the broad band at 16,330 cm^−1^. The peak at 1540 cm^−1^ could be due to the N−H stretching vibration [62].

### 3.3. Adsorption Tests

#### 3.3.1. Effect of Adsorbent Concentration on DB78 Dye Removal Efficiency

The concentrations of chitosan, nanocellulose, and CCMB-0.25:1 were varied to investigate their effect on direct blue 78 dye removal efficiency. It was found that, by increasing the adsorbents concentration (increasing of adsorption active sites), the equilibrium loading would decrease, and the removal efficiency would increase until reaching the maximum efficiency and then approximately reach a constant value. The experiments were conducted by varying the concentration of chitosan powder from 1 to 6 g/L, nanocellulose from 0.25 to 2 g/L, and CCMB-0.25:1 from 1 to 15 g/L on DB78 dye solutions with initial concentrations of 50 and 100 mg/L, at a fixed temperature, pH, stirring speed, and mixing time.

**Figure 10:**
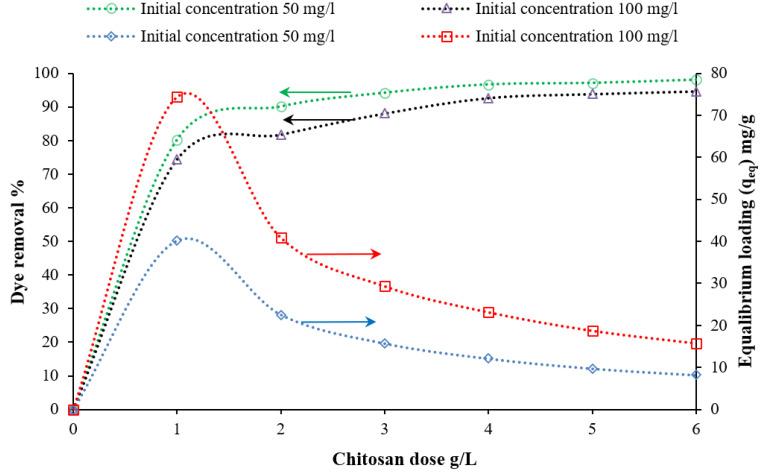
Effect of chitosan concentration on DB78 dye removal efficiency (temperature of 22 °C, pH of 8.5, mixing speed of 150 r.p.m., and contact time of 45 min).

For chitosan, it was observed from batch adsorption tests that a removal percentage of 94% can be obtained by using a chitosan dose of 3 g/L for a solution with an initial concentration of 50 mg/L, as shown in Figure 10. The maximum equilibrium loading reached was 74.4 mg/g.

**Figure 11:**
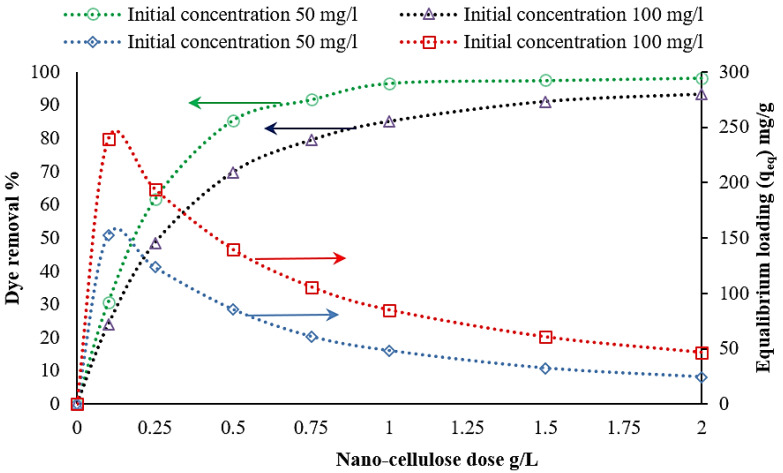
Effect of nanocellulose concentration on DB78 dye removal efficiency (temperature of 22 °C, pH of 2, mixing speed of 150 r.p.m., and contact time of 30 min).

For nanocellulose, it was observed that a removal percentage of 93.2% and equilibrium loading of 46.6 mg/g can be obtained by using a nanocellulose dose of 2 g/L for solutions with an initial concentration of 100 mg/L, as shown in Figure 11. The maximum equilibrium loading reached was 239 mg/g.

**Figure 12:**
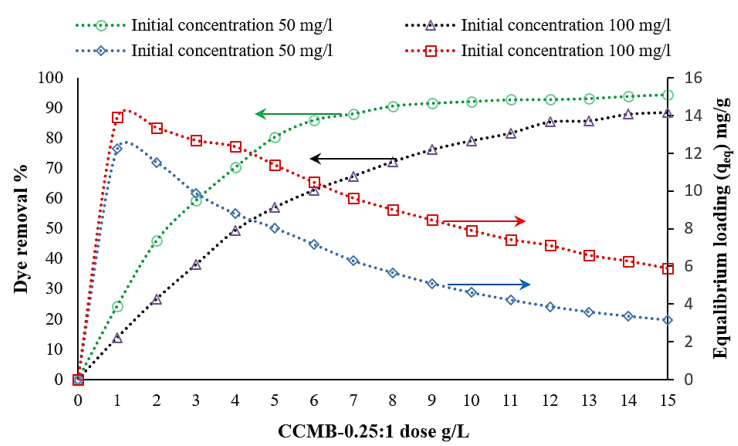
Effect of CCMB-0.25:1 concentration on DB78 dye removal efficiency (temperature of 22 °C, pH of 3.5, mixing speed of 150 r.p.m., and contact time of 120 min).

For CCMB-0.25:1, it was observed that a removal percentage of 92.1% and equilibrium loading of 4.6 mg/g can be obtained by using a CCMB-0.25:1 dose of 10 g/L for solutions with an initial concentration of 50 mg/L, as shown in Figure 12. The maximum equilibrium loading reached was 13.9 mg/g. Table 2 presents the optimal adsorbent concentration for direct blue 78 dye removal.

#### 3.3.2. Effect of Solution pH on DB78 Dye Removal Efficiency

**Figure 13:**
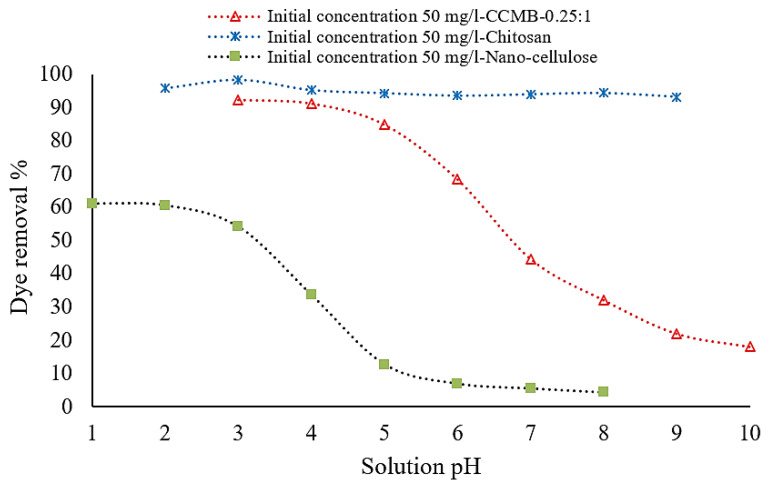
Effect of pH value on DB78 dye removal efficiency (temperature of 22 °C, pH of 1–10, and mixing speed of 150 r.p.m.).

The effect of the initial pH of dye solution was experimentally investigated under a pH range from 1 to 10, and the results can be observed from Figure 13. For chitosan, the solution pH has a little effect on chitosan adsorption behavior. Chitosan reaches its maximum loading (maximum removal efficiency of 98.3%) under acidic conditions (pH = 3), in comparison with a 93% removal efficiency under alkaline conditions (pH = 9).

This can be attributed to the presence of acidic conditions, where hydrogen ions (H^+^) could protonate the amine groups (–NH_2_) of chitosan.

Chitosan–NH_2_ + H^+^ → chitosan–NH_3_^+^ (3)

The direct blue 78 dye was dissolved in aqueous solution, and the sulfonate groups were separated and converted into anionic dye ions.

DSO_3_Na → DSO_3_^−^ + Na^+^ (4)

The adsorption process then ensued due to the electrostatic interaction between these two ions [63].

Chitosan–NH_3_^+^ + DSO_3_^−^ → chitosan–NH_3_^+^-O_3_SD (5)

The pH value is one of the most important process variables when considering dye adsorption. The adsorption of a positive charged adsorbate is favored when the pH of the solution is greater than the point of zero charge (pH_pzc_) of the adsorbent. The point of zero charge indicates that the net charge on the whole particle surface (i.e., the surface of the absorbent) is zero. Whereas the adsorption of negatives charges, in turn, is favored at pH levels less than pH_pzc_. Therefore, the adsorption of the anionic dyes is expected to be favored in solutions with pH values less than the pH_pzc_ of the adsorbent [64].

From this point, it can be concluded that the swelling of chitosan powder into chitosan beads in the presence of acidic conditions will protonate the amine groups (NH_2_) into NH_3_^+^. This process will improve the electrostatic interaction between chitosan particles and dye ions and enhance the chitosan ability for anionic dyes’ removal. The adsorption process was significantly improved in the acidic solution. Therefore, it is supposed that the adsorbent surface is positively charged, and this is favorable to the adsorption of anionic dyes. The enhanced electrostatic interactions were formed between the positively charged bioadsorbent’s surface and the negatively charged SO_3_ group of dyes in acidic solutions.

**Figure 14:**
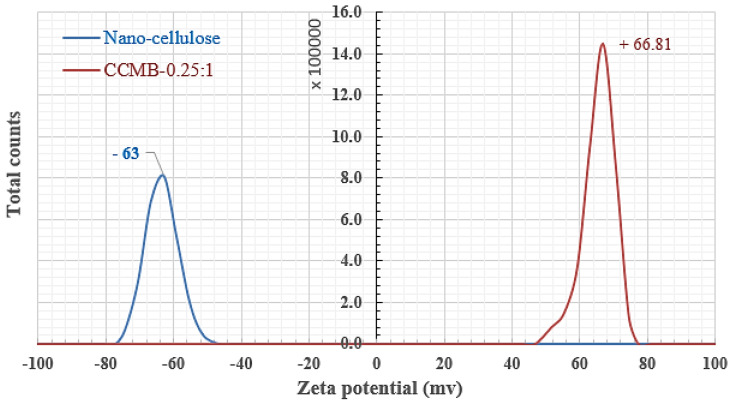
Zeta potential analysis of nanocellulose and nanocellulose/chitosan microbead (CCMB-0.25:1).

As seen in Figure 13, the initial pH of the solution has a higher effect on the direct blue 78 dye solutions’ removal process. Nanocellulose reaches its maximum adsorption capacity for DB78 dye under acidic conditions, i.e., a pH range from 1 to 2, and then the adsorption capacity is decreased sharply during the increasing of the pH from 3 to 6, and then it decreases slowly down to a pH equal to 8. Figure 14 shows that nanocellulose particles and CCMB-0.25:1 have a negative net surface charge (Zeta potential = −63 mv) and positive net surface charge (Zeta potential = +66 mv), respectively. The negative Zeta potential value of −63 mV is attributed to the presence of highly electronegative sulfate groups on the surface of the cellulose nanoparticles. The maximum adsorption capacity for DB78 dye was found in acidic conditions within a pH range from 3 to 4, and then it decreased sharply as the pH increased from 5 to 8.

#### 3.3.3. Effect of Mixing Time on DB78 Dye Removal Efficiency

The effect of the mixing time on both the percentage of DB78 dye removal and adsorbent loading was investigated. It is experimentally observed that the percentage of dye removal increased with the increasing mixing time, even reaching the optimal removal efficiency (equilibrium concentration, C_e_); at this time, the adsorbent is reaching its maximum loading capacity (equilibrium loading, q_e_).

**Figure 15:**
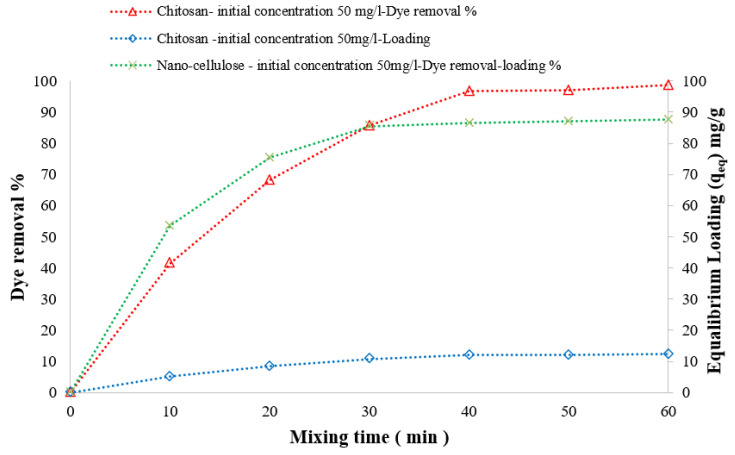
Effect of mixing time on DB78 dye removal efficiency. Adsorbents are chitosan and nanocellulose; temperature, 22 °C; pH, 8.5–2; and mixing speed, 150 r.p.m.

**Figure 16:**
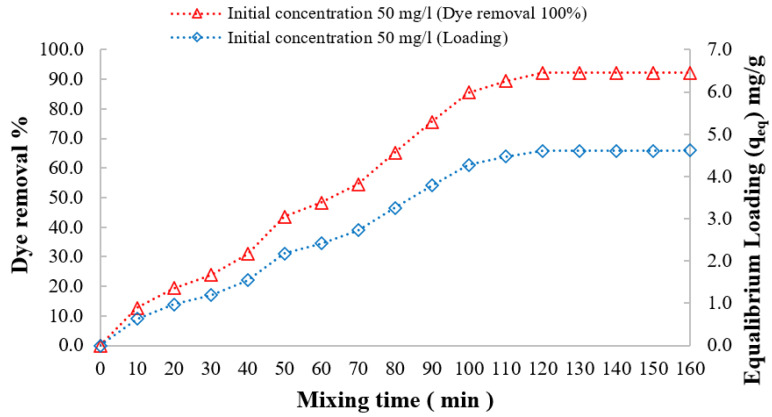
Effect of mixing time on DB78 dye removal efficiency. Adsorbent, CCMB-0.25:1; temperature of 22 °C; pH of 3; and mixing speed of 150 r.p.m.

For 45 min of contact time and a 4 g/L adsorbent dose of chitosan, the equilibrium concentration decreased to 1.6 mg/L, the dye removal efficiency was 96.7%, and the optimum loading capacity was 12.1 mg/g for an initial dye concentration of 50 mg/L solution. For 30 min of contact time and 500 mg/L as the adsorbent dose of nanocellulose, the equilibrium concentration decreased to 7.2 mg/L, the dye removal efficiency was 85.7%, and the optimum loading capacity was 85.5 mg/g for an initial dye concentration of 50 mg/L solution, as shown in Figure 15. For 120 min of contact time and 10 g/L as the adsorbent dose of CCMB-0.25:1, the equilibrium concentration decreased to 3.9 mg/L, the dye removal efficiency was 92.1%, and the optimum loading capacity was 4.6 mg/g for an initial dye concentration of 50 mg/L solution, as shown in Figure 16.

#### 3.3.4. Effect of Dye Initial Concentration Removal Efficiency

**Figure 17:**
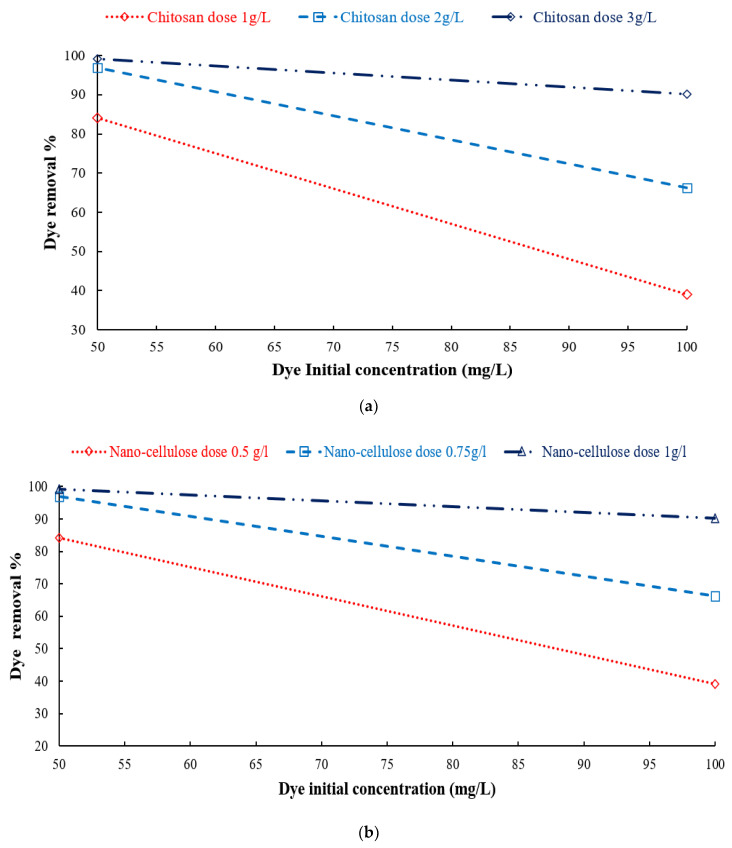

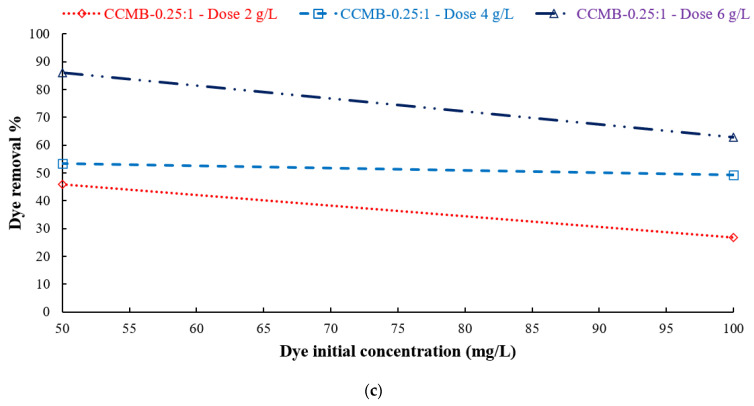
Effect of dye initial concentration on removal efficiency using different adsorbents: (**a**) chitosan, (**b**) nanocellulose, and (**c**) CCMB-0.25:1.

The direct blue 78 (DB78) dye solutions had initial concentrations of 50 and 100 mg/L. Figure 17a shows that the removal efficiency at 80.3% and 77.2% from using chitosan dose 1g/L for the initial concentrations 50 and 100 mg/L, respectively. The removal efficiency was improved to 90.1% and 84.6% by using chitosan dose 2 g/L. At a higher concentration of neat chitosan (3 g/L), the removal efficiency of the direct blue 78 (DB78) dye reached 94.2% and 90.8% at the initial dye concentration 50 and 100 mg/L, respectively.

Figure 17b shows that the removal efficiency was obtained at up to 85.4% and 69.7% for the dyes’ initial concentration of 50 and 100 mg/L, respectively, using 0.5 g/L of nanocellulose. A higher removal efficiency was observed when using 0.75 g/L nanocellulose. The removal efficiency of DB78 was found to be 91.7% and 79.4% when using 1 g/L of nanocellulose for initial concentrations of 50 and 100 mg/L, respectively. For CCMB-0.25:1 at a dose of 6 g/L, the removal efficiency was 86% and 63%, respectively, for the 50 and 100 mg/L initial concentrations of the dyes, as shown in Figure 17c. These results show that the adsorption process is highly dependent on the initial concentration of dyes.

#### 3.3.5. Effect of Nanocellulose Concentration on Removal Efficiency

**Figure 18:**
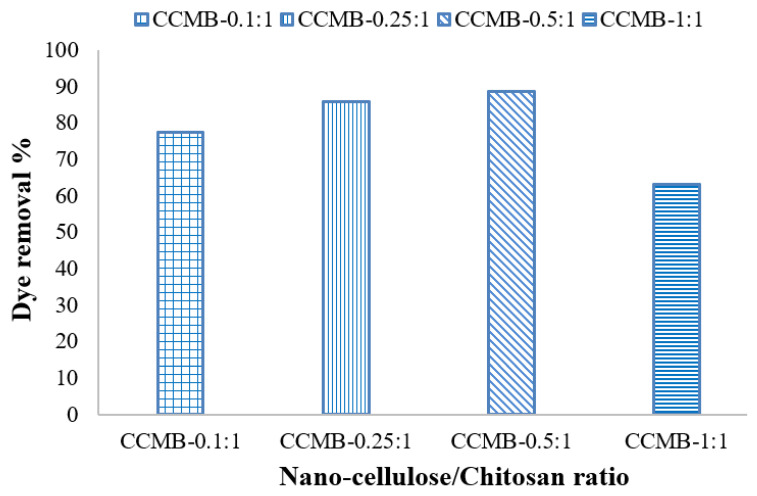
Effect of nanocellulose concentration in microbeads on removal efficiency.

To study the effect of nanocellulose loading using nanocellulose/chitosan microbeads (CCMBs) on dye-removal efficiency, a different ratios were applied: CCMB-0.1:1, CCMB-0.25:1, CCMB-0.5:1, and CCMB-1:1. The experimental results showed that the dye-removal efficiency increases with the increase nanocellulose loading, up to 0.5:1. At higher nanocellulose loads, the removal efficiency decreases, as shown in Figure 18. This can be attributed to blockage of internal porosities of chitosan by the incorporated higher nanocellulose loadings.

### 3.4. Adsorption Isotherm

**Figure 19:**
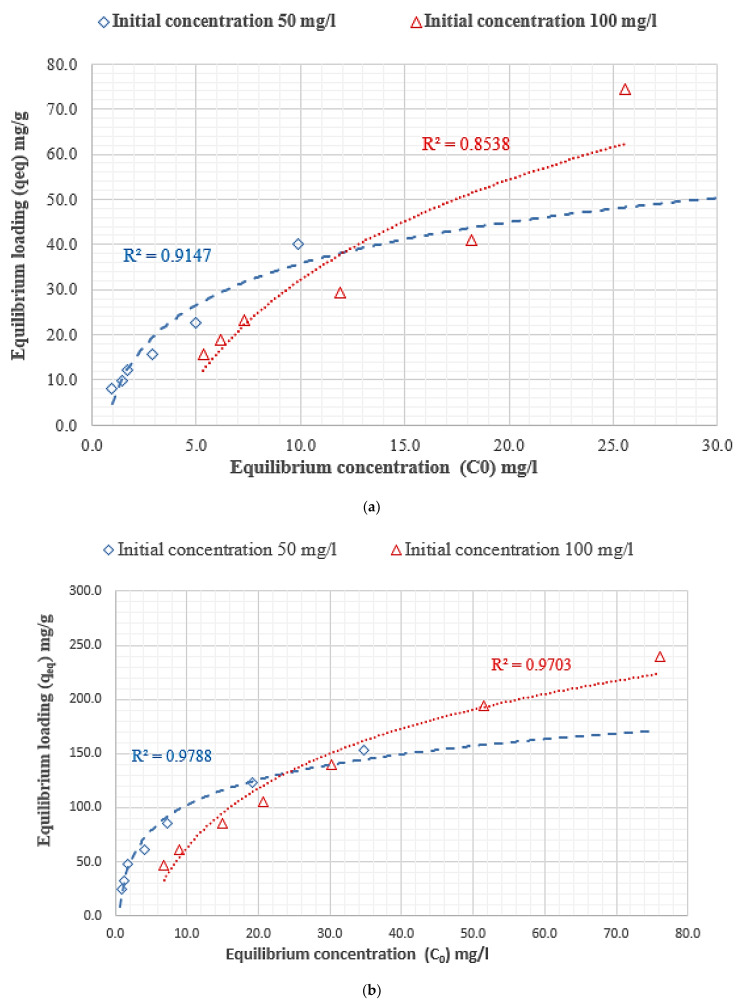

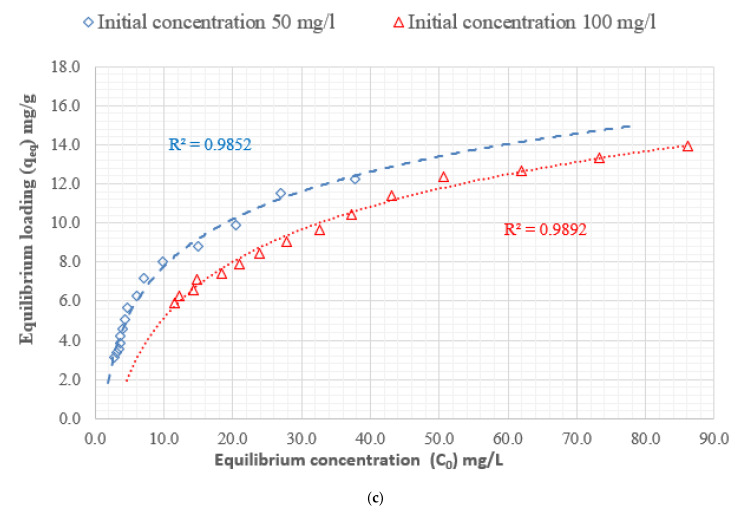
Adsorption isotherm for direct blue 78 dye removal using adsorbents (**a**) chitosan, (**b**) nanocellulose, and (**c**) CCMB-0.25:1.

As shown in Figure 19, the adsorption isothermal curve indicates the quantity of adsorbate DB78 dye that can be adsorbed by the adsorbents (chitosan, nanocellulose, and CCMB-0.25:1), q_eq_, in comparison to the adsorbate concentration in the liquid state (C_e_q). These are essential considerations in the design of adsorption systems. Moreover, the form of the equilibrium curve helps to describe other phenomena linked with the adsorbent–adsorbate interaction. The equilibrium curves are identified in four main classes, according to the primary slope, and the subgroups are described for each class based on the upper parts’ shapes and the slope changes: (a) S curves or vertical orientation isotherm, (b) L curves or normal or “Langmuir” isotherms, (c) H curves or high-affinity isotherms, and (d) C curves or constant partition isotherm [65].

The initial shape of the equilibrium curve (L shape) in Figure 19 follows the basic premise that, the higher the solute concentration, the greater the adsorption capacity, until the number of adsorption site clearance is limited, and competition occurs between the solute molecules for the available sites. This isotherm type indicates that the adsorption occurs due to relatively weak forces, such as “van der Waals forces”. There are several isothermal models (equations) available, and the two important isotherms are selected in this study, namely the Freundlich and Langmuir isotherms.

**Figure 20:**
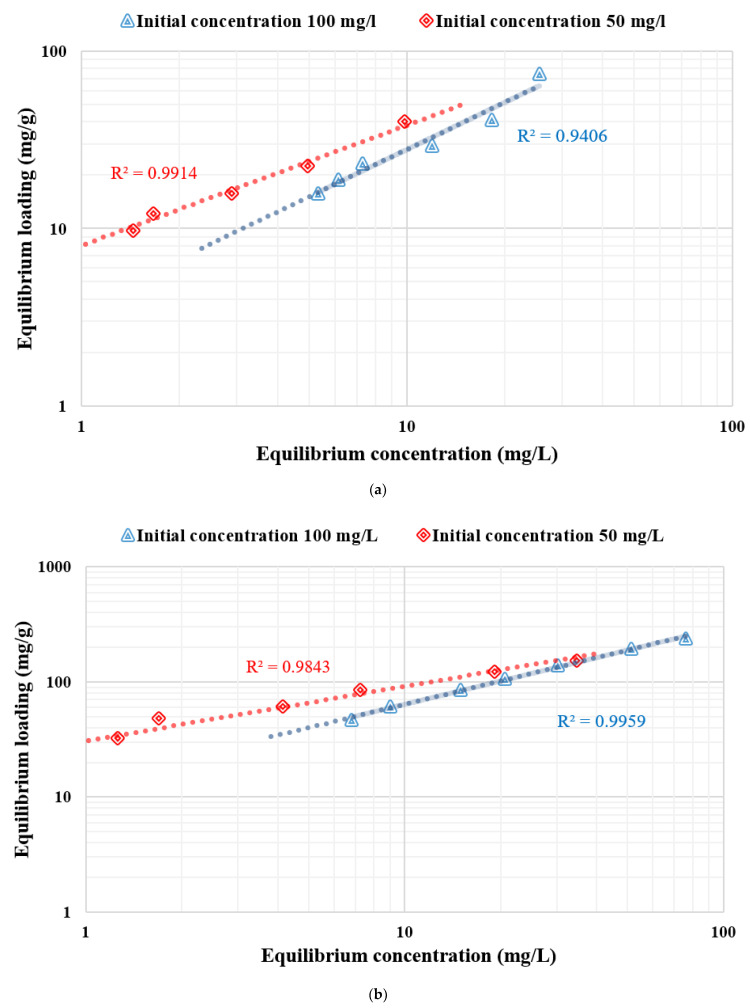

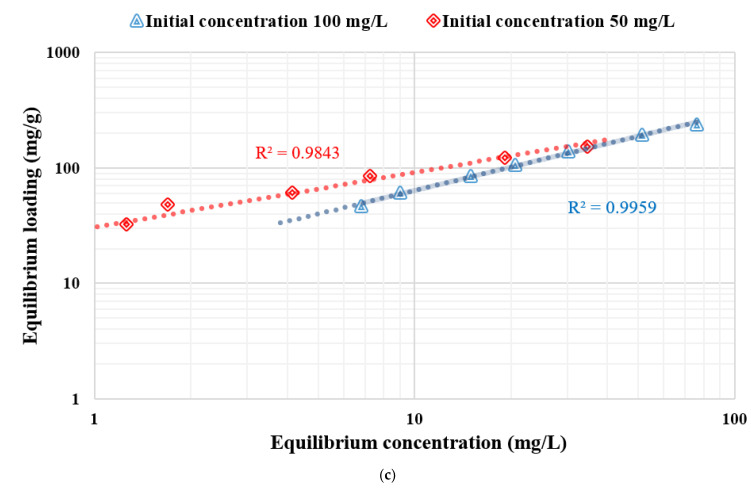
Freundlich adsorption isotherm for direct blue 78 dye removal using adsorbents (**a**) chitosan, (**b**) nanocellulose, and (**c**) CCMB-0.25:1.

The Freundlich isotherm believes that the adsorption happens on a heterogeneous surface, and the adsorbed mass increases exponentially with an increase in concentration [66]. This isotherm explains equilibrium on heterogeneous surfaces, and, hence, capacity is not presumed to be a monolayer. In liquid phase, this isotherm is given by Equation (6):

Q_e_ = K_f_ C_e_^1/nf^ (6)

where k_F_ is the Freundlich fixed value (k_F_ unit = mg/g, where c = 1/n_F_ is the heterogeneity factor). This isotherm focuses on integrating the role of adsorbent–adsorbate surface interactions. Figure 20 indicates the application of equilibrium data according to the Freundlich isotherm. For chitosan as an adsorbent, the Freundlich constant k_f_ values were 8.02 and 3.65, and the heterogeneity factor 1/n_f_ values were 0.67 and 0.88, respectively, for solutions with the initial concentrations of 50 and 100 mg/L. For nanocellulose as an adsorbent, the Freundlich constant k_f_ values were 30.6 and 13.49 mg/L, and the heterogeneity factor 1/n_f_ values were 0.47 and 0.67 for the initial concentration of 50 and 100 mg/L, respectively. For CCMB-0.25:1 as an adsorbent, the Freundlich constant k_f_ values were 2.18 and 2.12, and the heterogeneity factor 1/n_f_ values were 0.51 and 0.43 with the initial concentrations of 50 and 100 mg/L.

The Langmuir isotherm believes that sorption occurs within the adsorbent at different homogeneous sites, and it has been successfully applied to several processes of sorption. The isotherm’s physical simplicity is based on some assumptions: Adsorption cannot occur beyond monolayer coverage. Each site can hold only one adsorbate molecule. All sites are energetically equivalent, and the surface is uniform. The linear form of the Langmuir isotherm is given by Equation (7):

(C_e_/qe) = (1/Q_0_ b) + (C_e_/Q_0_) (7)

where C_e_ is the equilibrium concentration (mg/L), q_e_ is the mass adsorbed at equilibrium (mg/g), Q_0_ is the adsorbent loading (mg/g), and b is the adsorption energy (Langmuir fixed value L/mg). The values of Q_0_ and b were determined from the slope and intercept of the linear plots C_e/_q_e_ versus C_e_, resulting in a straight line of slope 1/Q_0_, corresponding to the total coverage of monolayer (mg/g), and the intercept is 1/Q_0_b [67,68].

**Figure 21:**
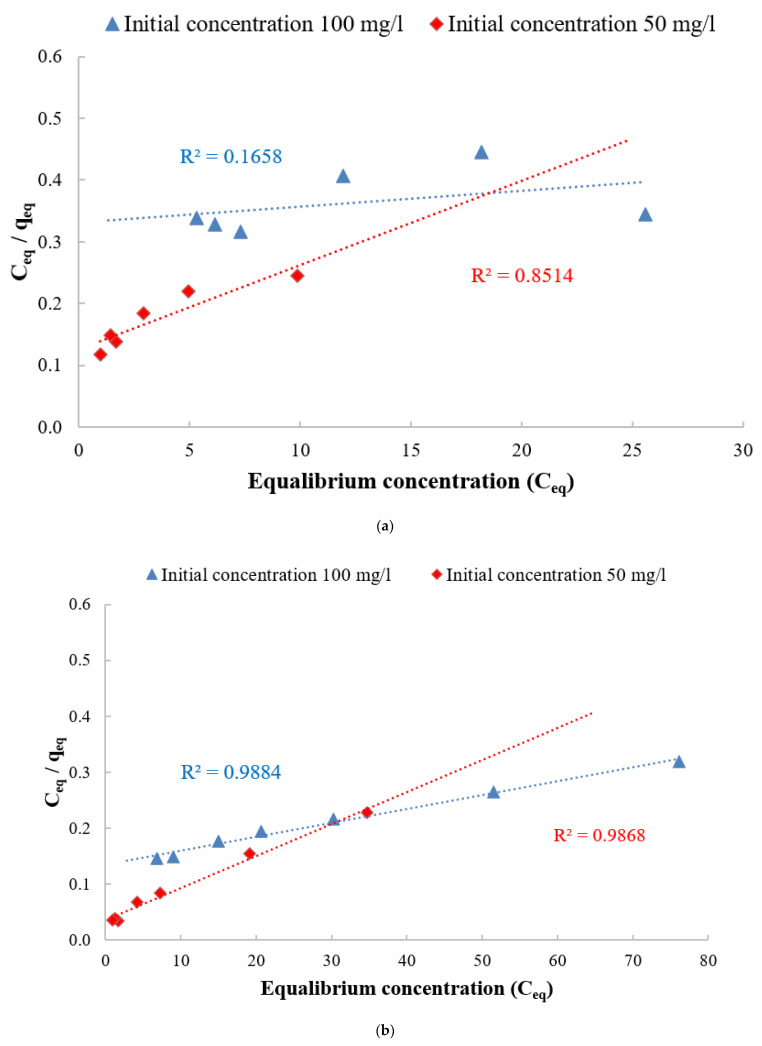

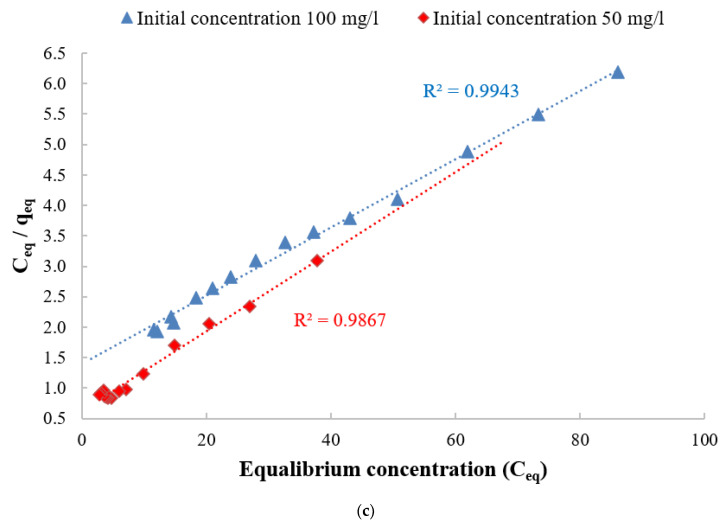
Langmuir adsorption isotherm for direct blue 78 dye removal using adsorbents (**a**) chitosan, (**b**) nanocellulose, and (**c**) CCMB-0.25:1.

Figure 21 indicates the application of equilibrium data according to the Langmuir isotherm. For chitosan, the adsorbent loading value (Q_0_) was 73.5 mg/g, and the Langmuir fixed value (b) was 0.107 L/mg for the initial concentration of 50 mg/L. For nanocellulose, the adsorbent loading value (Q_0_) was 175.4 mg/g, and the Langmuir fixed value (b) value was 0.16 and 1/mg. For CCMB-0.25:1, the adsorbent loading value (Q_0_) was 15.3 mg/g, and the Langmuir fixed value (b) was 1.03 L/mg.

It was observed from the listed adsorption isothermal models in Table 3 that they follow the Freundlich isotherm for chitosan as an adsorbent. The Langmuir isotherm is for CCMB-0.25:1 as an adsorbent, and both the Freundlich and Langmuir are for nanocellulose as an adsorbent.

### 3.5. Adsorption Kinetics

**Figure 22:**
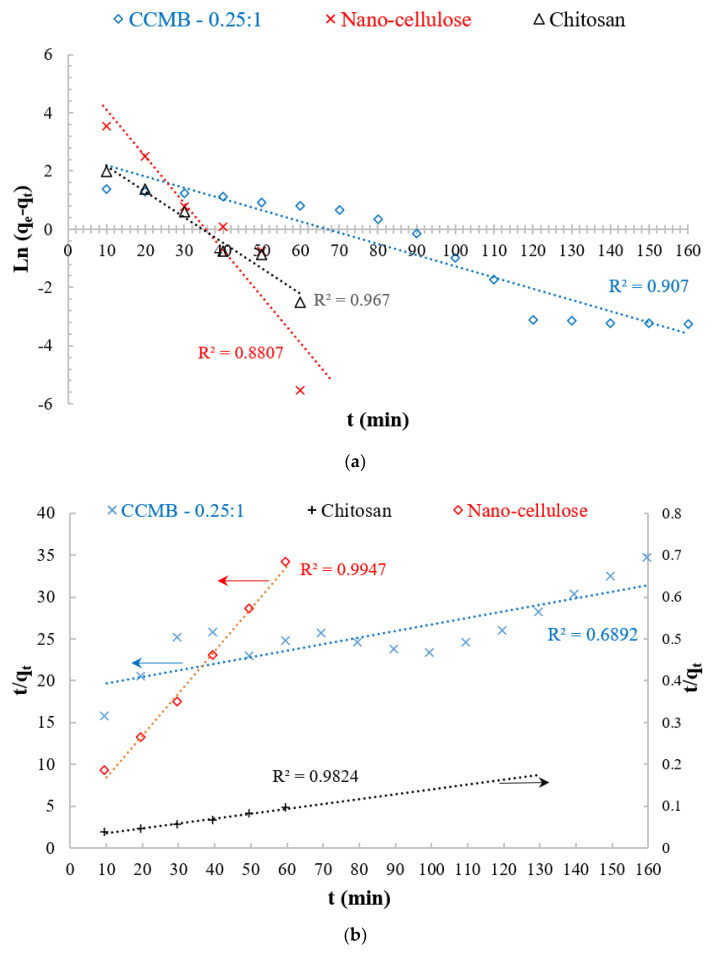
Adsorption kinetic studies: (**a**) pseudo first-order model and (**b**) pseudo second-order model.

In order to understand the mechanism of adsorption process, the kinetic studies were conducted by extracting and analyzing the samples at time intervals of 10 min until the consecutive residue dye concentrations became closer. The kinetic data for the adsorption process of DB78 dye onto chitosan, nanocellulose, and CCMB-0.25:1 with an initial dye concentration of 50 mg/L were examined with the well-known kinetic models, namely pseudo first-order model (PFO) and pseudo second-order model (PSO). The plotting of these kinetic models is shown in Figure 22.

Pseudo first-order equation:

The pseudo first-order kinetic equation was used for the adsorption analysis. The linear form of this equation is as follows:

ln (q_e-_q_t_) = ln q_e_–k_1_ t (8)

where q_e_ (mg/g) and qt (mg/g) are the amounts of adsorbed adsorbate at equilibrium and at time, t, respectively. K_1_ (min^−1^) is the rate constant of pseudo first-order model.

Pseudo second-order equation:

The adsorption kinetics can also be described by the pseudo second-order model. The linear form of the pseudo-second-order equation is expressed as follows:

(t/q_t_) = (1/k_2_q_e_^2^) + (1/q_e_) t (9)

where k_2_ (g/mg min) is the equilibrium rare constant of pseudo second-order adsorption; and q_e_ (mg/g) and qt (mg/g) are the amounts of adsorbed adsorbate at equilibrium and at time, t, respectively [69,70].

Figure 22 shows the linear plots of PFO and PSO models of CCMB-0.25:1, nanocellulose, and chitosan. The kinetic parameters are listed in Table 4. On the basis of the low correlation coefficient for PSO and the high value for PFO, the adsorption abilities of CCMB-0.25:1 follow PFO rather than PSO; on the other hand, the adsorption behavior of nanocellulose and chitosan follows PSO rather than PFO. These results suggested that, for CCMB-0.25:1, PFO can best predict the kinetic process. The value of q_e_ = 12.8 mg/g calculated by PFO was more similar to practical q_e_ = 11.5 mg/g than PSO. For nanocellulose and chitosan, PSO can best predict the kinetic process. The values of mg/g calculated by PFO were more similar to practical q_e_ = 11.5 mg/g than PFO, as shown in Table 4. For chitosan, the applicability of the PSO model indicates the interaction between dye molecules and amino groups. Hence, the adsorption system is chemical adsorption. It was reported that the adsorption process of DB78 dye onto chitosan is best fitted to pseudo second order with a chemical adsorption mechanism [71].

**Figure 23:**
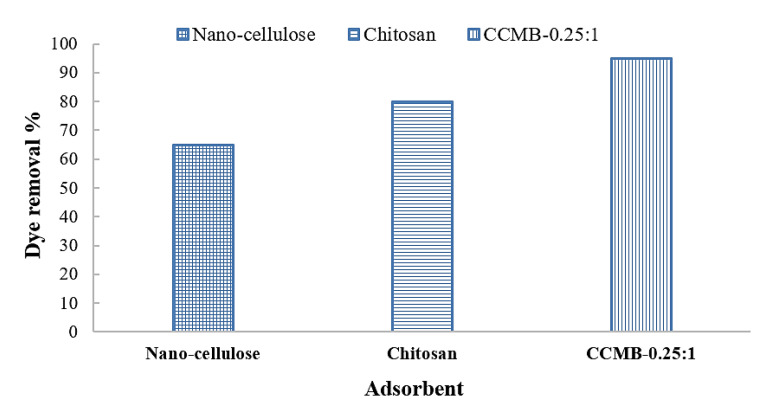
Effect of adsorbent type on dye removal efficiency.

The chitosan in powder form showed a high adsorption capacity, as shown in Figure 23; this capacity can be attributed to its high surface area, but it needs longer sedimentation time (8 h). For the chitosan microbeads formed, the swelling of chitosan powder into microbeads in the presence of acidic conditions improved its adoption capacity, due to the protonation of amine groups (NH_2_) into NH_3_^+^. This modification process led to a significant decrease in sedimentation time. Surface area of chitosan in the form of beads is less than the surface area of chitosan in powder form. Therefore, the chitosan microbeads were loaded with cellulose nanoparticles in order to improve the surface area and increase its adsorption capacity.

**Figure 24:**
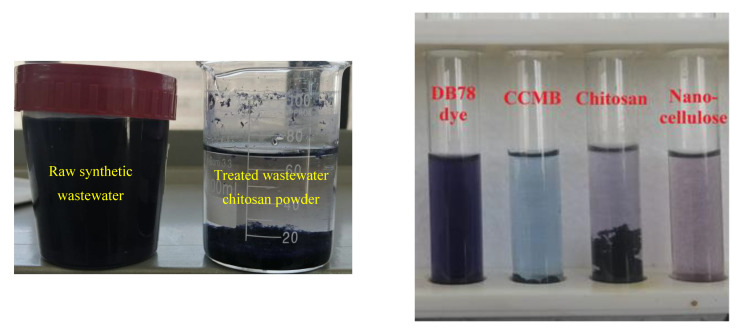
The adsorption process of DB78 dye by using adsorbents (chitosan, nanocellulose, and CCMB-0.25:1).

CCMB has shown a good adsorption capacity, in addition to remarkable short sedimentation time, with a low dose. Five minutes is sufficient to complete the sedimentation process. “Based on the experimental results, maximum removal efficiency 80% can be achieved using chitosan dose 1 g/L. While 65% removal was obtained using nano-cellulose dose 0.25 g/L. In the case of CCMB-0.25:1, 1 g/L of chitosan and 0.25 g/L of nano-cellulose can produce a microbead with a remarkable adsorption capacity of. Removal efficiency 95% with optimal dose 10 g/L was achieved using CCMB-0.25:1”, as shown in Figure 23. Figure 24 shows the adsorption process of DB78 dye, using adsorbents (chitosan, nanocellulose, and CCMB). It was observed that clear water can be obtained in the presence of chitosan powder after a long sedimentation time, i.e., 8 h. CCMB showed the lowest sedimentation time (5 min). Chitosan powder and cellulose nanoparticles showed longer sedimentation times than CCMB.

## 4. Conclusions

The removal of direct blue 78 dye (anionic) from single-component synthetic wastewater by adsorption with nanocellulose, chitosan, and novel nanocellulose/chitosan microbeads (CCMB) was experimentally investigated. An altered microbead with different nanocellulose/chitosan ratios (0.1:1, 0.25:1, 0.5:1, and 1:1) was synthetized in order to study the effect of nanocellulose dose on removal efficiency. The removal efficiency increases with an increasing nanocellulose dosage in synthetic microbeads up to a nanocellulose/chitosan ratio 0.5:1, and then the efficiency decreases with an increasing nanocellulose dosage. The adsorption process is highly dependent on the initial solution pH; the CCMB-0.25:1 reaches its maximum loading under acidic conditions (pH 3.5). It was observed from experimental studies that removal efficiencies of 94%, 91.7%, and 92.1% can be obtained by using the adsorbents chitosan, nanocellulose, and CCMB-0.25:1, respectively, for dye solution with an initial concentration 50 mg/L. Equilibrium studies have shown that the initial shape of the equilibrium curve is an L-shape, meaning that the adsorption process resulted from electrostatic interaction between dyes molecules and adsorbent particles (physical forces). Adsorption studies were modeled by using the Langmuir and Freundlich isothermal models. Therefore, chitosan, nanocellulose, and CCMB could be highly efficient sorbents for removing anionic contaminants. The abovementioned excellent performances of chitosan, nanocellulose, and CCMB demonstrated them as promising dye adsorbents for wastewater treatment.

## Figures and Tables

**Table 1:** The computation data resulted from the application Refine Version 3.0 Software Program (Kurt Barthelme’s and Bob Downs) for [Indol-4Ap]^TF^.

Symmetry Compound	2θ	d	hkl	Observed		Calculated		FWHM	D_av_
				2θ	2d	2θ	2d		
Parameters	15.453	5.7296	111	15.331	5.882	0	1.3 × 10^−5^	5.7296	83.04
*a* = 15.96 Å; *b* = 7.85 Å; *c* = 10.87 Å	20.332	4.4253	051	20.332	4.425	0	4 × 10^−6^	1.7565	270.86
*α* = *γ* = 90, *β* = 97.931°	22.655	3.9201	020	22.635	3.974	0	0	3.9201	121.37
V = 1400 (22), rmse ^(a)^ = 1.05 × 10^−3^	34.575	2.5920	23$\bar{2}$	34.575	2.613	0	−1 × 10^−6^	2.5920	183.56
Machine error = −0.48									
Average								3.5995	164.71

^(a)^ Root mean square error.

**Table 2:** Optimal adsorbent concentrations for direct blue 78 dye removal.

Adsorbent	Initial Concentration 50 mg/L			Initial Concentration 100 mg/L		
	Dose (g/L)	Loading (mg/g)	Removal %	Dose (g/L)	Loading (mg/g)	Removal %
Chitosan	3	15.7	94.2	5	18.77	93.85
Nanocellulose	1	48.3	96.6	2	46.6	935
CCMB-0.25:1	9	5.08	91.52	14	5.9	88.4

**Table 3:** Comparison of adsorption isothermal models for adsorbents (chitosan, nanocellulose, and CCMB-0.25:1).

Adsorbent	DB78 Dye Initial Concentration (mg/L)	Langmuir Isothermal			Freundlich Isothermal			Followed
		Q_0_ (mg/g)	b (L/mg)	R^2^	K_f_ (mg/g)	1/n	R^2^	
Chitosan	50	73.5	0.107	0.8514	8.02	0.67	0.9914	Freundlich
	100	384.6	0.0074	0.1658	3.65	0.88	0.9406	Freundlich
Nanocellulose	50	175.4	0.16	0.9867	30.6	0.47	0.9843	Langmuir
	100	400	0.018	0.988	13.49	0.67	0.9959	Freundlich
CCMB-0.25:1	50	15.31	1.0306	0.9867	2.18	0.51	0.9428	Langmuir
	100	17.79	0.0404	0.9943	2.12	0.43	0.9841	Langmuir

**Table 4:** Kinetic models parameters.

Kinetic Model Parameters	Pseudo First-Order Model			Pseudo Second-Order Model		
	K_1_ (1/min)	q_e_ (mg/g)	R^2^	K_2_ (g/mg min)	q_e_ (mg/g)	R^2^
CCMB-0.25:1	0.0385	12.8	0.907	0.0003	13.2	0.689
Nanocellulose	0.1594	292	0.8807	0.0015	99	0.9947
Chitosan	0.0867	20.9	0.967	2.807	17.27	0.9824

## Data Availability

Further data is available on request from the authors.
